# OncoDB: an interactive online database for analysis of gene expression and viral infection in cancer

**DOI:** 10.1093/nar/gkab970

**Published:** 2021-10-28

**Authors:** Gongyu Tang, Minsu Cho, Xiaowei Wang

**Affiliations:** Department of Pharmacology and Regenerative Medicine, University of Illinois at Chicago, USA; Department of Mechanical Engineering and Materials Science, Washington University in St. Louis, St. Louis, MO, USA; Department of Pharmacology and Regenerative Medicine, University of Illinois at Chicago, USA; Department of Pharmacology and Regenerative Medicine, University of Illinois at Chicago, USA; University of Illinois Cancer Center, Chicago, IL, USA

## Abstract

Large-scale multi-omics datasets, most prominently from the TCGA consortium, have been made available to the public for systematic characterization of human cancers. However, to date, there is a lack of corresponding online resources to utilize these valuable data to study gene expression dysregulation and viral infection, two major causes for cancer development and progression. To address these unmet needs, we established OncoDB, an online database resource to explore abnormal patterns in gene expression as well as viral infection that are correlated to clinical features in cancer. Specifically, OncoDB integrated RNA-seq, DNA methylation, and related clinical data from over 10 000 cancer patients in the TCGA study as well as from normal tissues in the GTEx study. Another unique aspect of OncoDB is its focus on oncoviruses. By mining TCGA RNA-seq data, we have identified six major oncoviruses across cancer types and further correlated viral infection to changes in host gene expression and clinical outcomes. All the analysis results are integratively presented in OncoDB with a flexible web interface to search for data related to RNA expression, DNA methylation, viral infection, and clinical features of the cancer patients. OncoDB is freely accessible at http://oncodb.org.

## INTRODUCTION

The advent of large-scale multi-omics datasets, such as those presented in The Cancer Genome Atlas (TCGA), have greatly helped us to understand the characteristics of a wide variety of tumors. With these cancer datasets, molecular aberrations at the DNA, RNA, protein and/or epigenetic levels can be systematically identified to better characterize human cancers. To date, TCGA is the most prominent cancer genomics program which offers >10 000 primary tumor and matched normal samples spanning 33 cancer types (1). TCGA hosts genomic and epigenomic data with clinicopathologic information, which provides a significant opportunity for data mining of cancer-relevant functional changes. However, the TCGA web portal provides few functions on statistical analysis or data visualization, and analysis of TCGA data can be difficult for most researchers who have limited bioinformatics skills. Thus, it is important to develop user-friendly online resources to analyze and visualize aberrant molecular changes in various types of cancer.

One important strategy to characterize cancer-relevant functions is to profile aberrant changes in tumor transcriptomes by RNA-seq analysis. Based on RNA-seq data, differential expression analysis is commonly performed to identify significantly altered genes between tumor and normal samples (2). Besides RNA-seq data, DNA methylation data are also very useful to identify aberrant epigenetic regulation of RNA transcription. For example, hypermethylation of TP53 gene leads to unchecked proliferation as well as inhibition of apoptosis in breast cancer (3).

Correlating transcriptomic and epigenomic data with clinical data can help identify aberrant molecular characteristics underlying disease development (4). Further, molecular biomarkers can be developed in this way for cancer diagnosis and prognosis. In particular, many RNA expression signatures have been reported for prediction of therapeutic response and survival outcome of cancer patients (5).

Besides aberrant DNA methylation or RNA expression, another major cause of cancer is viral infection. Human tumor-associated viruses (i.e., oncoviruses) are frequently identified as main drivers of cancer development (6). For example, HPV E7 oncoprotein could inactivate the tumor suppressor RB by disrupting the E2F-RB complex, triggering the degradation of RB via the ubiquitin-proteasome pathway (7). Thus, identification of oncovirus-related gene expression changes helps better understand the mechanisms underlying virus-induced cancers.

At present, multiple online resources have been developed for analysis of cancer genomic data by focusing on the TCGA datasets. One prominent tool is CBioPortal (8), which provides an interface for TCGA data download and visualization. However, CBioPortal is focused on tumor samples, and it does not include any data from normal samples for comparative analysis; in addition, there is no analysis functions on DNA methylation data. Another popular tool, GEPIA (9), is focused on RNA expression analysis using RNA-seq data from TCGA and GTEx (10), whereas MethHC (11) is a versatile web portal for epigenetic analysis using DNA methylation data collected from multiple sources. Although these are all very useful tools, to date, there is a lack of online resources to comprehensively study gene expression regulation in cancer by analyzing aberrant changes at both the RNA expression and DNA methylation levels. It is particularly important to further correlate molecular aberrations to clinical parameters to characterize cancer-relevant functional changes. Moreover, analysis functions on viral infection, a major cause of cancer, have not been systematically implemented in any online tool. To address these unmet needs, we established OncoDB, a comprehensive online database resource to explore abnormal patterns in gene expression as well as viral infection that are correlated to clinical features in cancer.

## METHODS AND RESULTS

### Data collection and processing

The datasets included in OncoDB are mainly from TCGA, encompassing RNA-seq, DNA methylation, and clinical data from over 9000 cancer patients. Specifically, for RNA expression analysis, we downloaded both tumor and matched normal RNA-seq data from the GDC data portal (https://portal.gdc.cancer.gov/). There is only a limited number of normal control samples in TCGA. To address this limitation, we also included RNA-seq data from over 1600 normal samples in GTEx (10,12). GTEx is a public resource to study tissue-specific gene expression among 54 non-diseased tissues.

**Figure 1.**
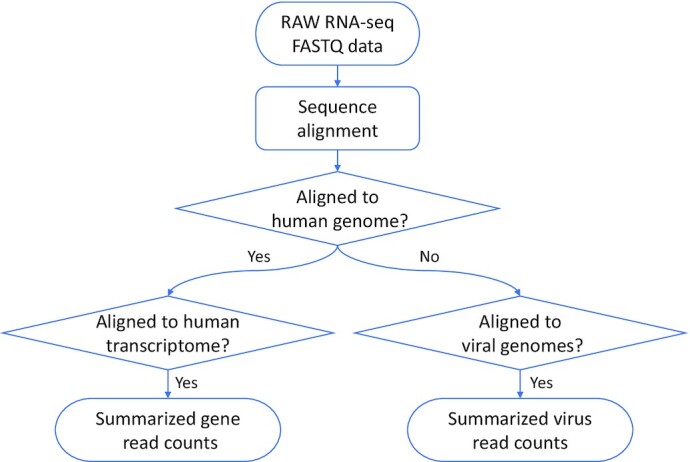
Workflow for the RNA-seq alignment pipeline to summarize human and viral sequencing reads.

We developed an RNA-seq data analysis pipeline, adopting standard features recommended by the GDC (https://docs.gdc.cancer.gov/). The workflow of our pipeline is outlined in Figure 1. In our pipeline, as the first step, both tumor and normal raw reads were aligned to the human genome with STAR (13). The aligned reads were further mapped to human RefSeq transcriptome to summarize raw gene-level read counts. On the other hand, for reads not aligned to the human genome, they were further aligned to all known human viral genomes presented in the RefSeq database (14) as well as the PaVE collection (15) to determine the virus status of each tumor. Raw gene-level read counts were further normalized using the transcripts per million (TPM) method. All TCGA and GTEx data were processed using the same bioinformatics pipeline. Of note, direct comparison of tumor and normal tissues may identify not only differences related to tumorigenesis, but also differences in tissue composition. Thus, the comparison results should be interpreted with caution.

Raw DNA methylation data were downloaded from the GDC data portal. Based on chromosome location annotations, we mapped individual methylation probes to all known genes in the human genome. The gene regions were defined from the RefSeq annotation file, while the promoter regions and transcription start sites were derived from Fantom5 (16). Major clinical parameters, including clinical stage, pathological stage, histological grade, and gender from all cancer types, were extracted from GDC flat files and further organized into structured data tables. The survival time and status were determined from curated patient follow-up data. The RNA-seq reads and DNA methylation profiles were mapped to clinical data based on de-identified patient ID.

### Development of the OncoDB database

All processed data described above were imported into a MySQL relational database. Server-side scripts written in Perl were implemented for data analysis and visualization. Statistical results and related figures were generated by Perl-CGI in conjunction with Python or R statistical program. In this way, we developed OncoDB, which is an online resource to analyze aberrant molecular changes in cancer. OncoDB provides multiple functions within four main modules, including RNA Expression Analysis, DNA Methylation Analysis, Clinical Analysis, and Oncovirus Analysis. In OncoDB, RNA expression and DNA methylation data are correlated with clinical data to characterize clinically relevant gene expression aberrations. As for the oncovirus module, gene expression and clinical data are analyzed in the context of viral infection to identify virus-related changes. The processed data can be retrieved through the data download interface. Details of the four OncoDB modules are presented below.

### Expression analysis

Dysregulation of RNA gene expression is a major cause of cancer. Thus, studying differentially expressed genes between tumor and normal samples helps infer cancer driver genes or potential therapeutic targets. Additionally, conducting correlation analysis among individual genes could help identify functional gene interactions in the gene regulatory network. In the RNA Expression Analysis module, three main features were implemented to enable statistical analysis and result visualization, including gene-focused expression analysis, cancer-focused expression analysis, and correlation of gene expression.

For these analyses, RNA-seq data are retrieved from the backend database and divided into two groups, tumor versus normal samples. Log_2_ fold change value is calculated between the two groups to determine whether a gene is upregulated or downregulated in tumor samples. Differential expression analysis is conducted with Student's *t*-test. A floor expression value of one is used to exclude genes with no or very low expression before performing Student's *t*-test. Pearson correlation analysis is employed to evaluate the correlation between two genes.

**Figure 2.**
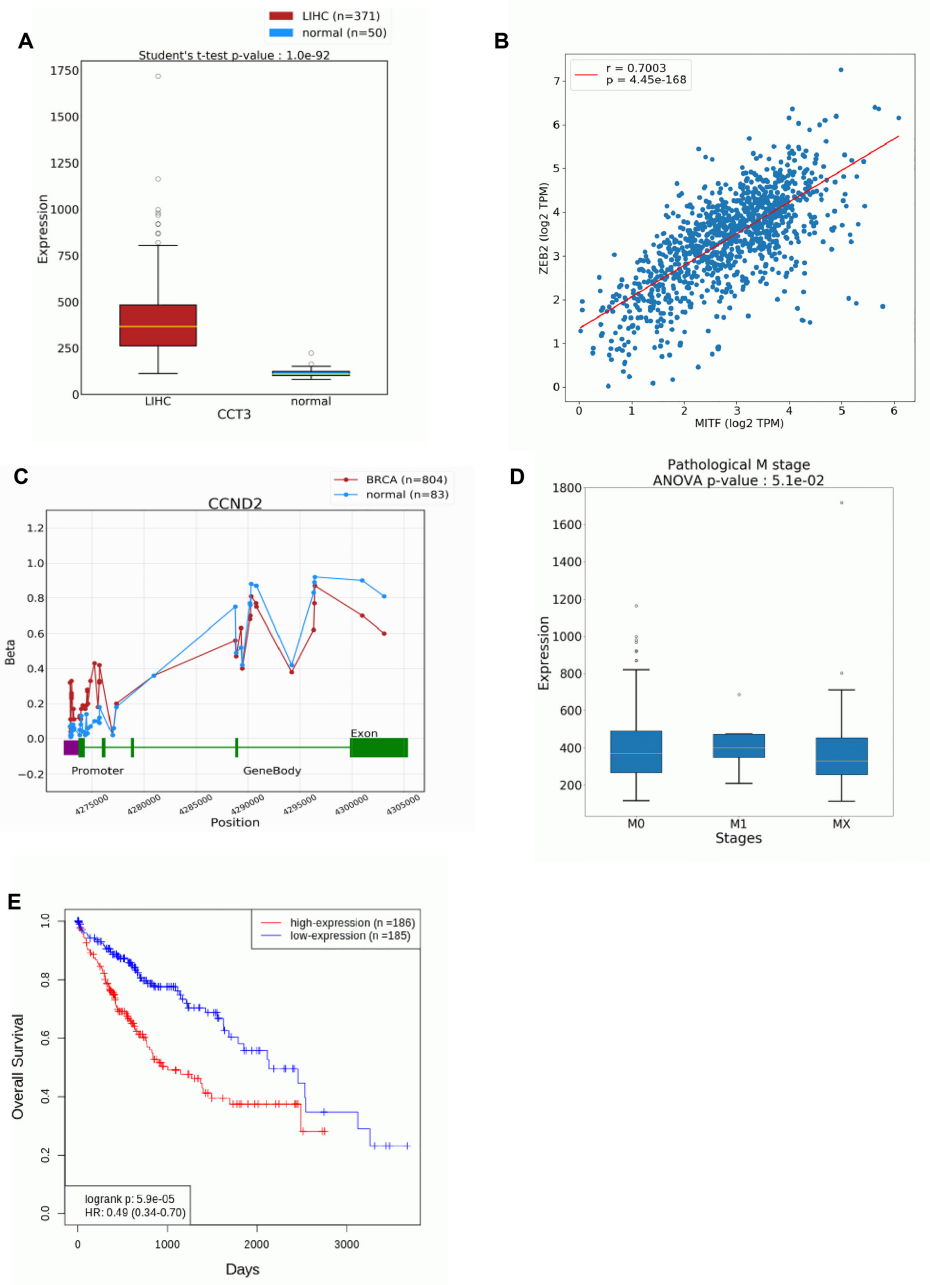
Examples to demonstrate features of the Expression Analysis, Methylation Analysis, and Clinical Analysis modules. (**A**) A boxplot to compare RNA expression level of CCT3 in liver tumor vs. normal samples. CCT3 was recently reported as a biomarker for liver cancer (17). (**B**) A scatter plot to compare RNA expression level of two genes (MITF and ZEB2) in breast tumor samples. Previous research indicates these two genes have highly correlated expression profiles (18). (**C**) A line graph to compare the DNA methylation level of CCND2 in breast tumor versus normal samples. Previous research reported that CCND2 is hypermethylated in breast cancer (19). (**D**) A boxplot for subgroup comparison of CCT3 RNA expression, stratified by pathological M stage in liver tumor samples. (**E**) A survival plot to evaluate the prognostic significance of CCT3 RNA expression in liver tumor samples.

A web interface was established to search for the expression profile of a user-specified gene in one or more cancer types. In addition, normal samples of the same tissue origin as the tumors are also analyzed to identify potential differences in gene expression profiles. The result is visualized in a boxplot (an example shown in Figure 2A) along with a table summarizing detailed statistical results. In addition, for any user-specified cancer type, OncoDB presents top-ranking differentially expressed genes between tumor and normal samples based on expression fold change and statistical significance. The result is retrieved and presented in a table for these genes, summarizing statistics such as false discovery rate (FDR)-adjusted *P*-value and log_2_ fold change. Besides making comparison between tumor and normal samples, for any selected cancer type, a user can also perform correlation analysis between two genes of interest. A scatter plot along with Pearson correlation analysis results are presented to compare two input genes (an example shown in Figure 2B).

### Methylation analysis

Increasing evidence shows that epigenetic dysregulation often results in aberrant RNA expression, and thus it is a common mechanism in cancer development. To this end, two main features were implemented in the Methylation Analysis module, including both gene-focused and cancer-focused analyses. Specifically, tumor samples and normal reference samples of the same tissue origin are compared to identify differential methylation patterns. Student's *t*-test is used in both gene-focused and cancer-focused methylation analyses. For a user-specified gene, differential methylation analysis is conducted for each probe individually to identify altered gene regions (including both the promoter and gene body). A line graph is presented to summarize averaged methylation levels for each probe within the gene structure (including the promoter, exon, and intron) for tumor or normal samples (an example shown in Figure 2C). In addition, probe-level analysis results are also summarized in a table, with statistically significant probes highlighted.

For a user-specified cancer type, there is an option to conduct differential methylation analysis for each gene using subsets of probes mapped to the promoter, gene body, or both. Differentially methylated genes are identified by comparing the average probe intensity of each gene between tumor and normal samples. A table is presented to summarize the statistics for top-ranking genes with differential methylation patterns.

### Clinical analysis

The Clinical Analysis module enables correlative analysis between clinical data and RNA expression / DNA methylation profiles. Specifically, clinical analysis can be performed by focusing on either user-specified gene or cancer type. Patient clinical data, including various clinical parameters, survival time, and outcome status, are retrieved from the backend database and associated with individual genes or cancer types. Clinical parameters consist of TMN stages and other common parameters such as age, alcohol consumption, gender, histology, smoking, BMI, family history, and race.

Analysis of Variance (ANOVA) test is performed to identify clinically significant genes by correlating RNA-seq or DNA methylation data to individual clinical parameters. When a user specifies a gene of interest for analysis, the result is displayed in a boxplot for RNA expression data or line graph for DNA methylation data (an example shown in Figure 2D). Moreover, an accompanying table is also presented to summarize analysis results including sample size, expression/methylation levels and statistical significance. On the other hand, when a user specifies a cancer type as well as a clinical parameter for analysis, the result table summarizes the statistics for top-ranking genes identified by their differential expression or methylation patterns associated with the selected clinical parameter and cancer type.

The survival plot feature presents the survival curves of user-specified gene in one or more specified cancer types, based on the Kaplan-Meier (KM) model which calculates the survival probability along with survival time. Based on the levels of RNA expression or DNA methylation, all selected cancer cases are stratified into either the high or low group according to a user-defined percentage cutoff. Additionally, summary statistics are presented, including the *P*-value from the log-rank test and hazard ratio from Cox proportional regression analysis. An example of the survival plot is shown in Figure 2E. In case the selected gene is significantly correlated with one or more clinical parameters, a table will also be presented to summarize the statistical analysis results.

### Oncovirus analysis

Viral infection is a major cause of cancer, leading to aberrant changes at various gene regulatory levels such as RNA expression and DNA methylation. Thus, differential expression / methylation analysis on virus-positive vs. virus-negative tumors can be a useful approach to identify viral targets in the human transcriptome, providing mechanistic insights into virus-induced cancers. In OncoDB, the oncovirus status of each tumor sample was determined with the sequencing analysis pipeline (Figure 1), and then used to stratify patient cases from user-specified cancer type. In this way, differential RNA expression or DNA methylation analysis can be performed to identify changes associated with viral infection.

**Figure 3.**
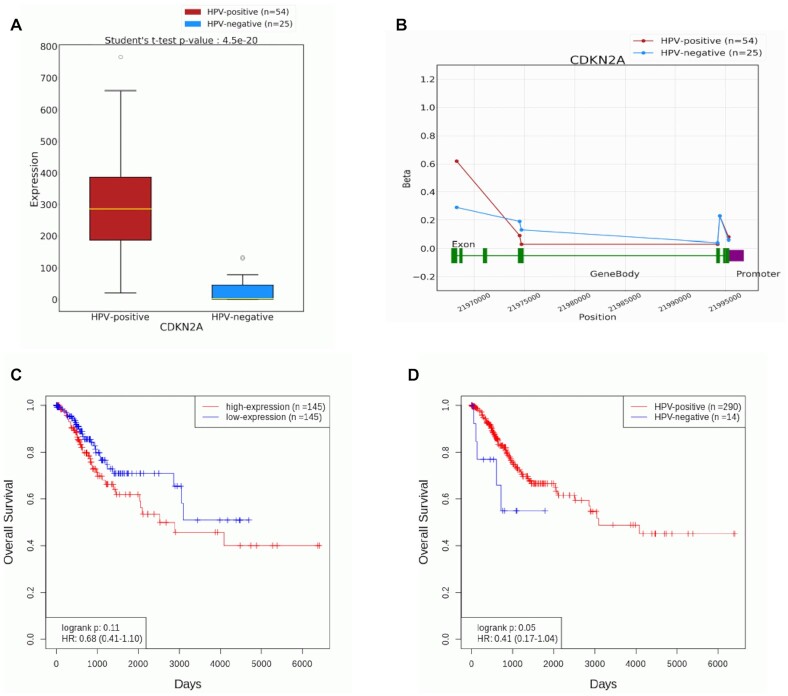
Examples to demonstrate features of the Oncovirus Analysis module. (**A**) A boxplot to compare RNA expression level of CDKN2A in HPV-positive vs. HPV-negative cervix tumor samples. CDKN2A (commonly known as p16) is a well-established marker for HPV infection (20). (**B**) A line graph to compare the DNA methylation level of CDKN2A in HPV-positive versus HPV-negative cervix tumor samples. (**C**) A survival plot to evaluate the prognostic significance of CDKN2A expression in HPV-positive cervix tumor samples only. (**D**) A survival plot to evaluate the prognostic significance of HPV infection in cervix tumor samples.

In the Oncovirus Analysis module, a user can perform gene-focused analysis to evaluate potential viral association for user-specified genes in one or more cancer types. A boxplot for expression analysis or a line graph for methylation analysis is presented to visually compare the virus-positive and virus-negative groups (examples shown in Figure 3A and B). In addition, differential analysis is performed to identify any statistical significance associated with the virus status, with the results summarized in an accompanying table. Alternatively, cancer-focused analysis can be performed to identify all top-ranking genes associated with user-specified virus and cancer type. In this case, a table is presented to summarize detailed statistical analysis results for all identified virus-associated genes.

The Oncovirus Analysis module also enables various virus-related gene analyses in the context of clinical data. For example, users can perform patient survival analysis to evaluate the prognostic significance of a gene (based on RNA expression or DNA methylation) in the virus-positive group or virus-negative group separately, with a survival plot presented for each group. An example of survival plot based on RNA expression analysis of CDKN2A in HPV-positive cervical cancer is shown in Figure 3C. Additionally, survival plots for cancer cases stratified by the virus status can be used to evaluate the prognostic value of user-specified virus in any specific cancer type. Figure 3D shows an example of survival plot grouped by the HPV status in cervical cancer. Users can also perform combined viral and clinical parameter analysis of a gene by specifying the virus, cancer type as well as clinical parameter of interest. In this case, the result is displayed in a boxplot for RNA expression data or line graph for DNA methylation data, stratified by user-specified clinical parameter for all virus-positive cancer cases. An accompanying table is also presented to summarize detailed analysis results such as ANOVA *P*-value to assess gene association with clinical parameters in virus-positive cases. Meanwhile, differential clinical analysis function provides a result table to summarize top-ranking genes with differential expression or methylation patterns based on user-specified virus, cancer type and clinical parameter. Lastly, users can analyze the expression of oncoviral genes from specific virus subtypes to correlate viral gene profiles with selected clinical parameters. The result is displayed in a boxplot along with a table to summarize detailed statistical results.

## CONCLUSION

OncoDB is a comprehensive online database to analyze gene expression and methylation data from over 10 000 patients spanning 33 major cancer types. Researchers could conveniently retrieve and analyze a large quantity of cancer data without requiring advanced bioinformatics skills. In OncoDB, RNA-seq, DNA methylation, and related clinical data are integratively analyzed and then visually presented on the web portal. Another unique feature of OncoDB is its oncovirus analysis module, which was developed by integration of viral infection status with cancer genomic and clinical data; this new feature provides functional as well as clinical insights into the roles of oncoviruses in cancer development and progression.

## DATA AVAILABILITY

All data in OncoDB are freely accessible at http://oncodb.org.
